# Structural analysis of polarizing indels: an emerging consensus on the root of the tree of life

**DOI:** 10.1186/1745-6150-4-30

**Published:** 2009-08-25

**Authors:** Ruben E Valas, Philip E Bourne

**Affiliations:** 1 Bioinformatics Program, University of California, San Diego, 9500 Gilman Drive, La Jolla, CA 92093, USA; 2 Skaggs School of Pharmacy and Pharmaceutical Sciences, University of California, San Diego, 9500 Gilman Drive, La Jolla, CA 92093, USA

## Abstract

**Background:**

The root of the tree of life has been a holy grail ever since Darwin first used the tree as a metaphor for evolution. New methods seek to narrow down the location of the root by excluding it from branches of the tree of life. This is done by finding traits that must be derived, and excluding the root from the taxa those traits cover. However the two most comprehensive attempts at this strategy, performed by Cavalier-Smith and Lake *et al*., have excluded each other's rootings.

**Results:**

The indel polarizations of Lake *et al*. rely on high quality alignments between paralogs that diverged before the last universal common ancestor (LUCA). Therefore, sequence alignment artifacts may skew their conclusions. We have reviewed their data using protein structure information where available. Several of the conclusions are quite different when viewed in the light of structure which is conserved over longer evolutionary time scales than sequence. We argue there is no polarization that excludes the root from all Gram-negatives, and that polarizations robustly exclude the root from the Archaea.

**Conclusion:**

We conclude that there is no contradiction between the polarization datasets. The combination of these datasets excludes the root from every possible position except near the Chloroflexi.

**Reviewers:**

This article was reviewed by Greg Fournier (nominated by J. Peter Gogarten), Purificación López-García, and Eugene Koonin.

## Background

There are two basic strategies for rooting the tree of life and defining the nature of the last universal common ancestor (LUCA), inclusive and exclusive. The recent argument for an Archaeal rooting [1] is inclusive and relies on arguments of primacy to establish the root. We feel such arguments often lead to circular reasoning based on one's expectation of what primitive cellular life should look like. For instance if one assumes that cellular life began in hydrothermal vents systems (reviewed in [2]) then one could argue that any organisms living in or near hydrothermal vents could be LUCA-like. But this does not prove that cellular life started in that condition. And even if it did, it is possible that later organisms invaded that niche, so the extant organisms there today are nothing like LUCA. Paralog rooting, where one uses paralogous sequences as an outgroup in a sequence tree [3,4], is technically an inclusive method since it attempts to determine which groups of sequences are the most primitive. However, this method is not self consistent [5,6] and technical objections have been raised [7].

Exclusive rooting defines branches as derived and thus they are omitted until only the root is left, thereby establishing LUCA. Ideally these two strategies would converge, but at this point there is no consensus even within one particular strategy as there are multiple ideas on the nature of the most primitive cellular systems [8,9] as well as how to properly exclude the root from a particular branch [7,10].

One method for arriving at an exclusive solution is top-down rooting using indels [10-13]. Usually an indel is ambiguous: it could be an insertion or a deletion. But if one knows the ancestral state the indel is polarized. That is, one can say which forms of the indel are the ancestral and derived states. One can then exclude the root from any branches where all the organisms have a derived form of the gene. One can infer the ancestral state of an indel by comparing a pair of paralogous genes that were duplicated before LUCA. Traditionally this technique would require a paralog set to be ubiquitous. Otherwise one could not be sure the paralogs diverged before LUCA. The advantage of top down rooting over traditional indel polarization is the ability to handle non-ubiquitous genes by considering gene loss and invention as well as insertion and deletion when analyzing the most parsimonious scenario for the history of a paralog set.

Lake *et al*. have presented 8 indel polarizations (summarized in [14,15]). They conclude the root of the tree of life lies between two clades. The first is the Actinobacteria (single membrane bacteria) and Gram-negatives (which Lake *et al*. refer to as double membrane bacteria). The second is the Firmicutes and Archaea (both of which contain single membranes). The authors have presented indels that apparently exclude the root from each of these clades so they conclude the root must lie between them. We present evidence using the addition of protein structure data that implies this conclusion is not supported.

There are several prerequisites for an indel argument to be correct in polarizing the tree of life. First, one needs a set of nearly universal paralogs (at least universal to the taxa being rooted). Second, one needs a quality alignment between those paralogs. This is often difficult as paralogs duplicated before LUCA have billions of years to drift and are under different selective pressures. The conclusions reached rely heavily on the alignment and this is the Achilles' heel of indel polarization. Where protein structures exist they offer the opportunity to get past the limitation of sequence drift, since structure is more conserved than sequence over long evolutionary time scales, and hence provides strong evidence when aligning proteins that diverged before LUCA. We introduce structural information into Lake *et al*.'s analysis where possible.

Cavalier-Smith has presented 13 polarizing transitions using a variety of data to reach an exclusive solution [7]. These transitions include information from indels, quaternary structures, as well as cellular organization. It is difficult to do his analysis justice in a short summary but here are the main points of his argument. He excludes the root from the Archaea and Eukaryotes based on proteasome evolution. He argues the transition from a Gram-positive membrane structure to a Gram-negative membrane would be much more difficult than the other direction, so this excludes the root from the single membrane prokaryotes. The Chloroflexi have the simplest known outer membrane, lacking outer membrane protein 85 (OMP85). OMP85 is present across all other gram-negative taxa so Cavalier-Smith places the root within or next to the Chloroflexi. He present other arguments as well that resolve the structure of the rest of the tree of life.

Despite reaching different conclusions about the root, both Lake *et al*. and Cavalier-Smith agree the root must lie within the Bacteria by excluding the root from the Archaea and Eukaryotes. Both agree that the Archaea must be derived from a Gram-positive bacterium, but Cavalier-Smith argues it was an Actinobacteria and Lake *et al*. argue it was a Firmicute. The arguments for each seem sound; both groups probably contributed genes to the Archaeal ancestor. The difference between which are the result of vertical versus horizontal transfer is not yet resolved in our opinion. The two methods also agree that the root is not within the Actinobacteria or the Firmicutes. It is important to realize that despite claims that all rootings of the tree are contradictory [16], the newest exclusive methods are converging on a Bacterial rooting. The most important thing these two bodies of work agree on is that there is a single backbone to the tree of life that can be resolved using rare events in evolution despite recent claims by the sequence tree community that no such tree exists or can be built [17,18]. However, the apparent disagreement between these datasets weakens the position that a single tree of life exists. We believe this work supports the idea that a backbone to the tree of life can be resolved and rooted by showing their analyses converge on the same result.

This work will focus on the fundamentally different conclusions about where the root of the Bacteria lies. Cavalier-Smith argues for a Gram-negative root based on his ideas about cells having an inside out origin, obcells [19]. He argues it would be easier for a Gram-positive cell to evolve by simply losing the outer membrane than it would be for a Gram-positive cell to gain an outer membrane and the cellular machinery needed to make it functional. The idea of a Gram-positive root is compatible with several scenarios for the origins of cells as well [20,21]. This argument will probably not be resolved on the basis of which theory of the origin of cells is more elegant since most of the ideas of early cellular evolution are highly speculative. Instead continued polarization of the transition between Gram-negatives and Gram-positives will lead to an understanding of which of these scenarios is even plausible.

We believe the indel in GyrA robustly excludes the root from the Actinobacteria using sequence alone [11] and there is no need to invoke structural alignments. However, we will subsequently present structural alignments, as well as other data, that support the exclusion of the root from Archaea based on insertions in elongation factors [12] since objections to these conclusions have been raised [1,22]. Lake *et al*. present 3 polarized indels that they claim exclude the root from the Gram-negatives: HisA (*P*-ribosylformimino-AICAR-P-isomerase), Hsp70 (heat shock protein 70 aka DnaK), and PyrD (dihydroorotate dehydrogenase). We will present evidence to suggest that none of these arguments truly excludes Cavalier-Smith's rooting. The Eobacteria (Cavalier-Smith's term for Deinococcus-Thermus and Chloroflexi) have the ancestral form of HisA despite being Gram-negatives. The conclusions about Hsp70 are based on a sequence alignment artifact, which is evident when a structural alignment is used instead. The arguments made by Lake *et al*. using PyrD are not self consistent, so we polarize this indel using quaternary structure. This excludes the root from the Archaea and Firmicutes, and probably from their last common ancestor as well. We also discuss the insert in Ribosomal Protein S12, which would have the potential to exclude Cavalier-Smith's rooting, but does not.

## Results

### Indels in elongation factors place the root within Bacteria

Several objections have been raised against the exclusion of the root from Archaea based on indels in the paralogs of initiation factors (IF) and elongation factors (EF) [1,22]. They claim the conclusions reached in [12] are based on alignment artifacts. These indels would be ideal to analyze using structure since they narrow the root to a single superkingdom. Di Giulio criticizes the alignment between EF-G and EF-Tu because there is a 4 residue stretch between the insertions that is more similar between some paralogs than between some orthologs [22]. Di Gulio is correct in raising a red flag here; there is probably an artifact in the sequence alignment. However, we argue the exclusion of the root from the Archaea is still valid in spite of that artifact.

**Figure 1:**
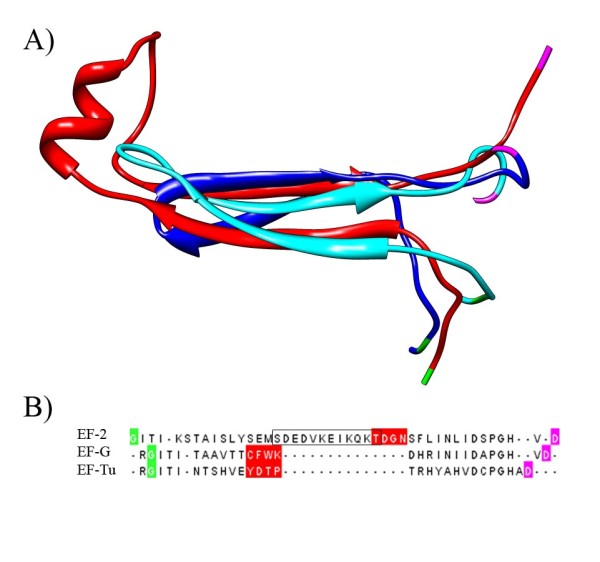
**EF-2 contains a derived insert**. A) Structural alignment of EF-G from *Thermus thermophilus *(2BV3 61–89 colored blue), EF-Tu (1EFC 58–86 colored cyan) from *Escherichia coli*, EF-2 from *Saccharomyces cerevisiae *(1N0U 67–110 colored red). B) Sequence corresponding to the structural alignment in A. The well conserved glycine and aspartic acid are highlighted green and magenta respectively in both the sequence and structure to show the disordered nature of this region. The 4 positions highlighted in red are aligned in the original alignment, which is why the alignment was critiqued in [22]. The additional insert in Eukaryotes relative to the Archaea is boxed in black.

Unfortunately the crystal structure of EF-2 (the Archaeal and Eukaryal orthologs of EF-G) from the Eukaryotes have a large disordered (unresolved) region near the indel of interest, hence these proteins are less suited for a structural alignment than the ones discussed below. The multiple structure alignments of these 3 regions is of poor quality due to the disordered region, which can be seen in the differing positions of the highly conserved residues on each end of this region (glycine colored green and aspartic acid colored magenta in Figure 1). However, the middle of the alignment seems reasonable and supports an insertion in EF-2 at the root of the Archaea.

We counted the distance between the well conserved RG(IV)T and PGH motifs in all elongation factors to reach a stronger conclusion (Table 1). Some sequences lack these motifs, but it is strongly implied they were present in the ancestral elongation factors since they are conserved across paralogs. The motifs are 20 residues apart in every EF-Tu and EF-1 sequence that have the motifs. The majority (55.95%) of EF-G sequences have the motifs 20 residues apart. This may actually be an underestimate because the next most populated length of 27 resides (32.16%) are mostly from β and γ-proteobacterial sequences. According to the Genomes Online Database [23], of the 1000 completed genomes published as of May 2009, 64 are β-proteobacterial and 215 are γ-proteobacterial genomes. These groups are over sampled relative to many others which would deflate the true proportion of the EF-G sequences that lack an insertion relative to EF-Tu. Even so, the most parsimonious ancestral elongation factor would have 20 residues between these motifs. Every Archaeal sequence that has the motifs in EF-2 has them 24 residues apart. Therefore, regardless of the actual alignment there must be a 4 residue insertion somewhere in EF-2 of the Eukaryotes and Archaea. Therefore the conclusion that the root can be excluded from the Archaea [12] is correct even though there is a sequence alignment artifact.

**Table 1:** Length between motifs in elongation factors.

	Total sequences	Do not have perfect match to motifs	% of sequences that have motifs	Length of region between motifs	Sequences with that length	% out of sequences with motifs
Ef-Tu	1013	12	98.82%	20	1001	100.00%
EF-1	53	3	94.34%	20	50	100.00%
EF-G	816	160	80.39%	20	367	55.95%
				24	13	1.98%
				25	22	3.35%
				26	2	0.30%
				27	211	32.16%
				28	12	1.83%
				29	11	1.68%
				31	9	1.37%
				33	4	0.61%
				35	3	0.46%
				40	1	0.15%
EF-2	52	9	82.69%	24	43	100.00%

A summary of the length of the region between the well conserved RG(IV)T/PGH motifs. This data implies the Archaeal ancestor of EF-2 had a 4 residue derived insertion regardless of which alignment is used.

The region of the indel in IF-2 using EF-G/Ef-2 as an outgroup examined in [1] is also in a region that does not align well structurally. Its sequence anchors are also much less conserved than in the indel discussed above, so the critique of this indel may be correct. However, the strength of this indel polarization appears to be a moot point. To the best of our knowledge no one has argued against Cavalier-Smith's exclusion of the root from Archaea based on proteasome structure [7,24], which is strongly supported by our own conclusions on proteasome evolution [25]. That taken with the derived insertion in EF-G and the quaternary structure of PyrD (discussed below) there are 3 strongly polarized arguments that each place the Archaea as derived from the Bacteria. To the best of our knowledge there is not a single argument that excludes the root from all the Bacteria in the same way these 3 polarizations exclude the root from the Archaea. Therefore the goal of the rest of the analysis of the indel polarizations is to narrow the root within the Bacteria.

### HisA and HisF exclude the root from all Gram-negatives except the Eobacteria

**Figure 2:**
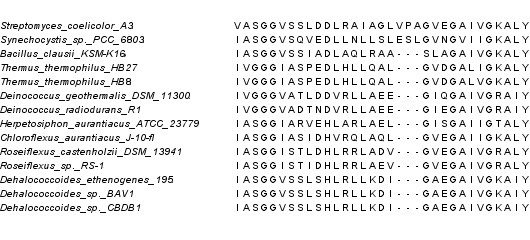
**HisA does not exclude the root from the Eobacteria**. A MUSCLE based alignment of all the HisA sequences in Eobacteria. Represenatives from Actinobacteria (*Streptomyces coelicolor A3*) and other Gram-negatives (*Synechocystis sp. PCC 6803*) are included to show the indel. All the Eobacterial sequences share the relative deletion with the Firmicutes (*Bacillus clausii*).

HisA and HisF are an ideal paralog set because they are nearly ubiquitous and have a relatively high degree of sequence similarity among paralogs. A structural alignment of the 3 forms of this indel reveals that the conclusions based on sequence alignments are valid (data not shown). Lake *et al*. conclude this excludes the root from the Actinobacteria and Gram-negatives [14]. However, their own summary of the indel shows the insertion that is present in most Gram-negatives is apparently absent in a *Deinococcus *genome. A realignment of just a few species that have the insert with the Eobacteria shows that all 11 fully sequenced Eobacterial genomes have a deletion relative to the other Gram-negatives (Figure 2). This means that the indel in HisA actually excludes the root from the Actinobacteria and all Gram-negatives except the Eobacteria. Cavalier-Smith claims the Eobacteria are some of the most ancient bacteria because they lack lipopolysaccahride in their membranes. The fact that HisA does not exclude Cavalier-Smith's root would not matter on its own, because two other indels apparently exclude the root from the Eobacteria. But we will argue neither of these arguments holds water, and that the results of Lake *et al*. and Cavalier-Smith converge on a rooting within the Eobacteria.

### Protein structure alignment renders the Hsp70/MreB indel inconclusive

Lake *et al*. claim that the Hsp70/MreB indel excludes LUCA from the Gram-negative bacteria [10]. This is not a new idea, and was first proposed by Gupta 10 years ago [26]. Hsp70 contains a large indel between the Gram-positives and Gram-negatives. Since Hsp70 is nearly universally distributed, if one can deduce the ancestral state of Hsp70 it would reveal which group is ancestral and which is derived. There is no indel between MreB and Hsp70 from the Gram-positives in Gupta's alignment. He argues the Gram-negatives are derived since they have an apparently derived insertion in Hsp70. However, Philippe has made the argument that Mreb and Hsp70 are very distant paralogs, so it is difficult to align them [27]. In his alignment it is not clear whether or not the Gram-positive form of Hsp70 has an insertion relative to Mreb. He raises the possibility that there are actually two independent insertion-deletion events. The newer work on this indel has dealt with the issue of the gene being missing in some species, but has not significantly improved the quality of the alignment [10].

**Figure 3:**
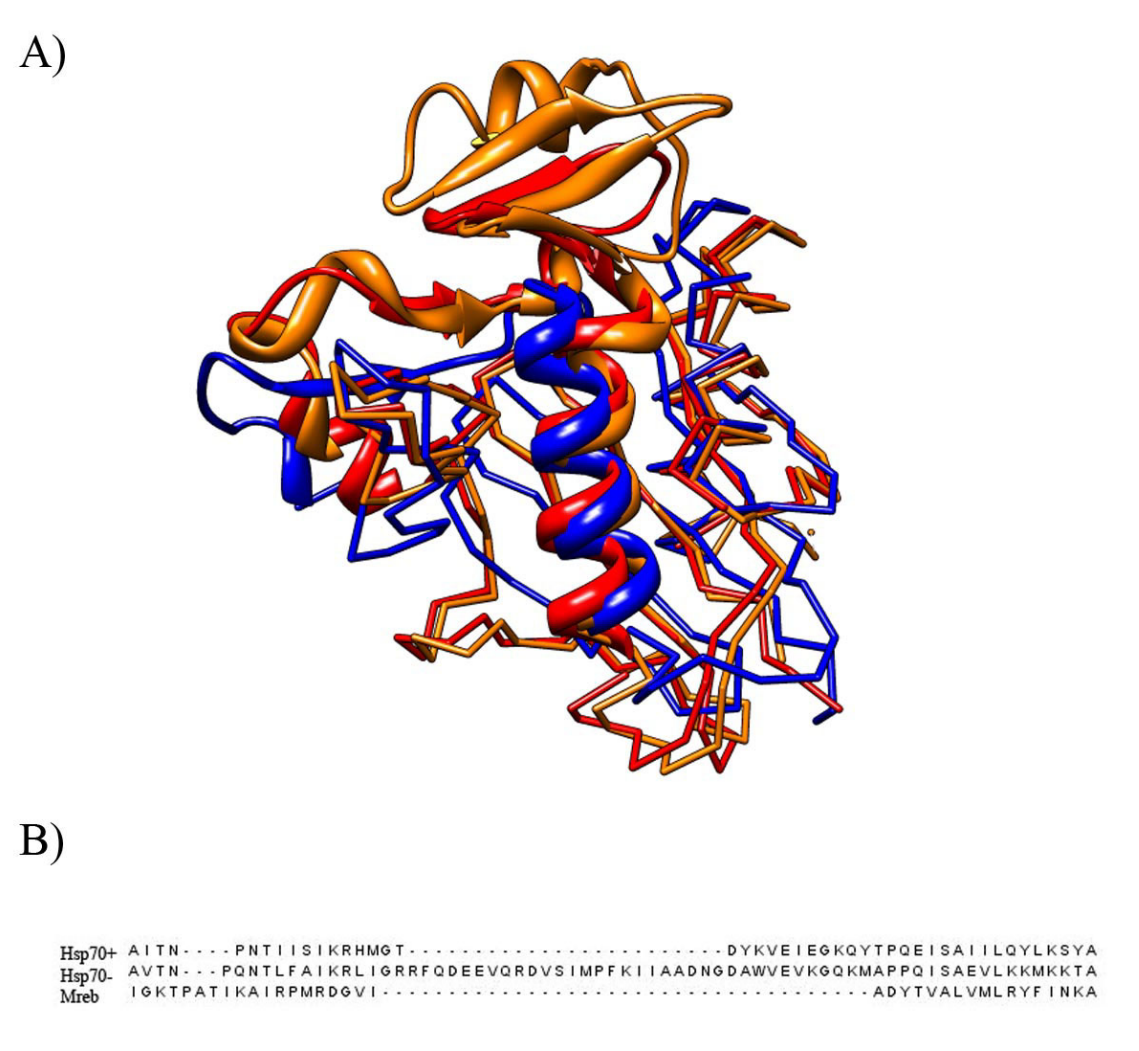
**Structural Alignment of MreB/Hsp70**. A) A multiple structural alignment of the MreB/Hsp70 C-terminal actin-like ATPase domain. The region around the indel is highlighted as a ribbon diagram. The backbone of the rest of the domain demonstrates high conservation between these structures. The blue chain is MreB from *Thermotoga Maritima *(1JCF:A 51–86 drawn as ribbon). The red chain is Hsp70 from the Gram-positive bacterium *Geobacillus Kaustophilus *(2V7Y:A 57–101 drawn as ribbon). The orange chain is Hsp70 from the Gram-negative bacterium *Escherichia coli *(1DKG:D 56–125 drawn as ribbon). B) The sequences corresponding to the highlighted portion of the structure alignment in A.

The recently solved crystal structure of a Gram-positive Hsp70 from *Geobacillus Kaustophilus *provides an opportunity to review the Hsp70/MreB situation using a structural alignment [28]. Structures of Hsp70 from both Gram-positive and Gram-negative bacteria were aligned with MreB using the CE-MC webserver [29]. These structures align well, which is expected since they are all in the same SCOP superfamily [30]. It is implied that Hsp70 from Gram-positives aligns perfectly to Mreb in this region [10,26,31]. A review of the structural alignment reveals this is not the case. Rather Hsp70 from Gram-positive bacteria have an insertion relative to Mreb (Figure 3). There has to be 2 independent insertion-deletions events here to account for the 3 different structures seen in this region. Therefore, it is impossible to determine the ancestral state of Hsp70. Every scenario requires two insertion-deletion events regardless of the root, and therefore this indel cannot be used to polarize the transition between Gram-positive and Gram-negative bacteria.

### Quaternary structure of PyrD excludes the root from the Archaea and Firmicutes

Lake *et al*. have polarized an indel in PyrD using HemE (uroporphyrinogen decarboxylase) to exclude the root from the Archaea and Firmicutes [13]. Later they polarized the same indel in PyrD using HisA and HisF as outgroups to exclude the root from the Gram-negatives and the Actinobacteria [14]. With these conclusions one could root the universal tree of life by polarizing the PyrD indel alone. This appears to be supported by the indels in HisA and Ribosomal Protein S12, but as discussed above and below, respectively, the conclusions Lake *et al*. reach on these 2 indels are also in question. We argue there is a contradiction in the analysis of PyrD, and propose an argument based on quaternary structure to resolve this contradiction.

All of the most parsimonious rootings with a HisA (or HisF) outgroup have the ancestral state of PyrD being a deletion relative to the derived state [14]. The authors consider this result independently of their results with HemE outgroups. However, the ancestral state of PyrD should be the same regardless of the outgroup. All of the trees that are the most parsimonious with the HemE outgroup imply the ancestral state of PyrD was an insertion relative to the derived state [10]. It is impossible for any one rooting to be the most parsimonious with both HemE and HisA as outgroups.

There are two possible sources of the contradiction. The first is an alignment artifact. Our structural alignment of PyrD, HisA, and HisF is in agreement with Lake *et al*.'s sequence alignment (data not shown). HemE appears to be more distant in structure and sequence to these other 3 proteins than they are between themselves. The structure alignments between PyrD and HemE are not consistent. They vary greatly depending on which structures are used. The alignment in [13] is between the 3rd beta sheet in HemE and the 7^th ^beta sheet in PyrD. These regions are technically homologous because this fold arose through a series of internal duplications [32], but the fact these are different regions within paralogous structures indicates this alignment should not be used for polarizing the indels. The duplication between the paralogs is more recent than the duplication between the subbarrels, so one should be aligning the same region of the structures between paralogs. If we assume their sequence alignments to be correct then the other possible source for the contradictory conclusions is convergent evolution. There are several variants of PyrD and HemE at this site which include an additional 1 residue indel. This implies this region of PyrD is tolerant to small indels, so convergent evolution at this site is not out of the question. Top-down rooting excludes all trees that are not the most parsimonious, which assumes there was no convergent evolution. In this case there is evidence for convergent evolution so top-down rooting should not be applied to this indel set.

**Figure 4:**
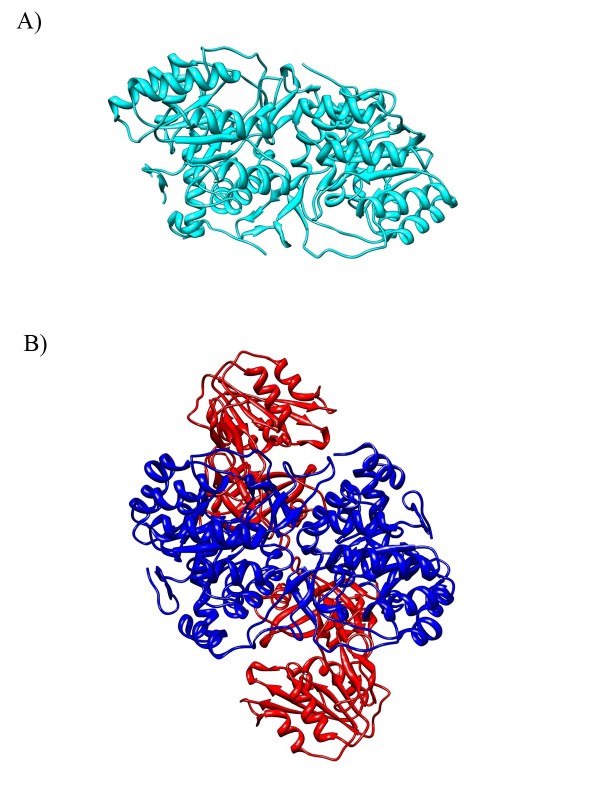
**Quaternary Structure of PyrD**. A) PyrD 1A from *Lactococcus lactis *is a homodimer (1JUB colored cyan). B) PyrD from *L. lactis *1B is a heterotetramer. The homodimer interface at the center of PyrD 1B (1EP3 colored blue) is similar to the interface in PyrD 1A. PyrD 1B has an additional subunit PyrK (colored red). This implies that PyrD 1B is derived from PyrD 1A.

Since the indel arguments contradict themselves, it is worth considering another line of reasoning. Lake *et al*. do not consider the quaternary structure of PyrD. There are 3 families of PyrD, each with a different solved quaternary structure [33]. The distribution of each family was examined using the NCBI Protein Clusters Database [34]. PyrD 2 (PRK07565) is a membrane bound monomer and is found across the Gram-negatives and Actinobacteria. PyrD 1A (PRK02506) is a homodimer that is mainly found within the Lactobacillales. PyrD 1B (PRK07259) is a heterotetramer found across the Archaea and Firmicutes (except in *Staphylococcus *that have PyrD 2). It has an extra subunit, PyrK. The core of this enzyme is a homodimer that is similar to PyrD 1A [35] (Figure 4). PyrD 1B has a deletion relative to the 2 other subfamilies. This deletion is polarized as the ancestral state when HisA or HisF are used as an outgroup, but is derived when HemE is used as an outgroup.

We argue the most parsimonious route for quaternary structure evolution would be monomer -> homodimer -> heterotetramer. A new protein-protein interface evolves at each step in this scenario. One can imagine a scenario where protein-protein interfaces are lost at each step but this requires a heterotetrameric ancestor. None of the other known structures in PyrD's or PyrK's superfamilies bind each other, which means there is no outgroup that makes a heterotetrameric ancestral state seem plausible. HemE is a homodimer and HisA is a monomer, so if either of these are the true ancestor of PyrD it does not make sense for the homotetramer to be the ancestral state. HisF is a heterodimer, but the other subunit appears to be unrelated to PyrK. Without a heterotetrameric outgroup the only way PyrD 1B could be ancestral is to have gained PyrK at the root of the PyrD tree. However, this subunit would have to be lost in PyrD 1A, so this is obviously not the most parsimonious scenario. The most parsimonious scenario for quaternary structure evolution is the one described above, and that excludes the root from Firmicutes and Archaea as well as their last common ancestor. Even if evolution was not completely parsimonious in this case it does not negate our polarization that places PyrD 1B as the derived state. We argue that independent insertion events in this region are more probable than the homotetramer being the ancestral structure. At the very least, PyrD should be considered inconclusive for excluding the root since the sequence and structure arguments disagree. PyrD is another structural argument that the Archaea must be derived from the Gram-positives in line with previous arguments on proteasome evolution [7,24,25].

**Figure 5:**
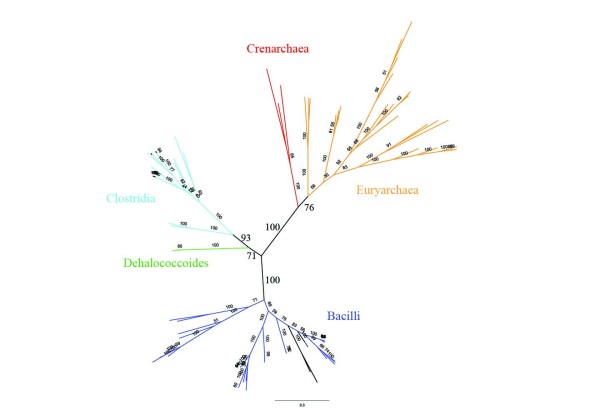
**Maximum likelihood tree of PyrD 1B**. Each of the major groups is separated by significant bootstrap values which indicates the distribution of PyrD 1B cannot be due to recent horizontal transfer.

A maximum likelihood tree for PyrD 1B has good separation between the Firmicutes and Archaea (Figure 5). This implies this distribution is not the result of horizontal transfer, but rather each of these groups ancestrally had a derived form of the protein. The Crenarchaea and Euryarchaea each cluster separately too. The Archaeal ancestor probably had PyrD 1B, but it was lost in several Crenarchaea. It must be noted that PyrD 1B is present in the *Dehalococcoides *(a subgroup of Chloroflexi). Based on their position in the tree this could be a horizontal transfer from the Firmicutes. However, even if the *Dehalococcoides *invented PyrD 1B its presence across a single genus does not exclude the root from the Chloroflexi.

### Ribosomal protein S12 and RpoC are probably not paralogs

Indel polarization requires special attention to the choice of paralogs. It has been argued that the indel in ribosomal protein S12 can be polarized using RpoC (DNA-directed RNA polymerase subunit beta') [13]. The authors claim this excludes the root from the Firmicutes and Archaea. This apparently derived insertion is present in all the Chloroflexi, which is not discussed by the authors. Ribosomal protein S12 belongs to the "OB-fold" in SCOP. RpoC belongs to the fold "beta and beta-prime subunits of DNA dependent RNA-polymerase". The overall structure of these proteins is different enough they are considered to be different folds. This alone is enough evidence that a sequence alignment between these proteins is probably meaningless. However the authors only claim the regions around the indel are homologous. They calculate an e-value of .002 that these 30 residues are paralogous in both proteins. This e-value is much worse than that of their other paralogs pairs (by up to several orders of magnitude). It is possible for there to be homology between proteins at a subdomain level as discussed in our recent review [36], but we see no evidence of that in this case. A pairwise alignment between ribosomal protein S12 (1J5E:L) and RpoC (2A69:D), both from *Thermus thermophilus*, was performed using FATCAT [37]. The regions in the sequence alignment do not align in the structure alignment at all. FATCAT concludes these structures are not similar (P-value of 9.96e-01). None of this is evidence these regions can be considered paralogous. It is possible these two regions do share a common ancestor, but since their structural context has changed it does not make sense to align their sequences. This raises the question of whether a sequence alignment can ever be considered meaningful without structural conservation. We conclude the indel in ribosomal protein S12 cannot be polarized using this out group. A structure search of the Molecular Modeling Database [38] revealed that no solved structures are homologous to ribosomal protein S12 in the region of interest despite their being many structures in the same family in SCOP. This indel probably cannot be polarized properly, and is not evidence against a root within the Chloroflexi.

## Discussion

**Figure 6:**
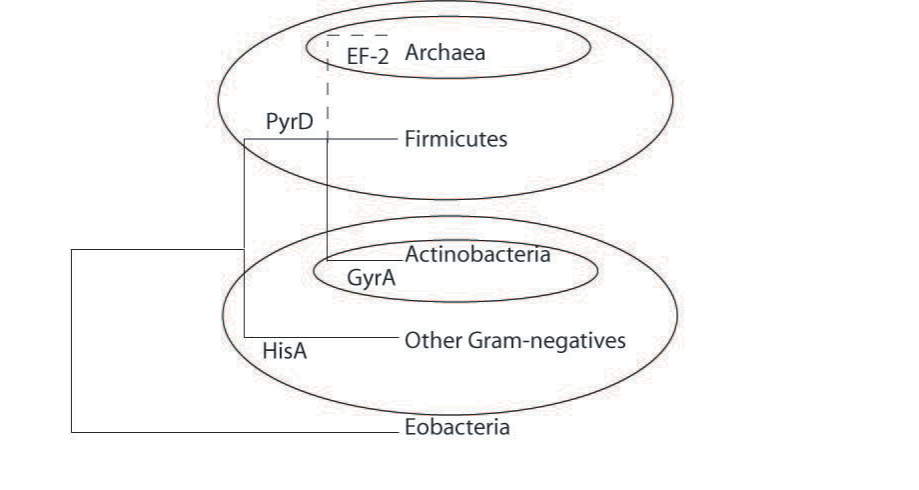
**Summary of data**. Each circle corresponds to an argument presented above that excludes the root of the tree of life from a particular branch. The Archaea are placed with the Gram-positives, but drawn with a dashed line because we do not wish to argue which Gram-positive group was their ancestor at this time.

There are four conclusions of Lake *et al*. that would disprove the rooting of the tree of life proposed by Cavalier-Smith if they are correct. We show that all four of these arguments have flaws and that is evidence that Cavalier-Smith's rooting is probably correct. The fact that the HisA indel excludes all the Gram-negatives except the Eobacteria is certainly a novel piece of evidence that supports the rooting within the Eobacteria. There are only a limited number of paralogs sets that are ubiquitous enough to be useful for rooting the tree. These sets will probably be exhausted without truly contradicting the Eobacterial root. Indel analysis reliably excludes the root from the Archaea, Actinobacteria and all Gram-negatives except the Eobacteria (summarized in Figure 6). Our polarization of PyrD's quaternary structure excludes the root from the Archaea and the Firmicutes, and their last common ancestor. If we combine these new interpretations of the indel data with Cavalier-Smith's 13 polarizing arguments there are no contradictions. All of this data supports the notion that LUCA must be near the Chloroflexi.

One of the major unresolved questions about LUCA is whether it had a DNA or RNA genome [39]. We argue that if LUCA was Chloroflexi-like, then it might have had a u-DNA genome. Thymidylate synthase is an essential enzyme that catalyzes formation of dTMP. There are two unrelated enzymes that perform this function, ThyA and ThyX [40]. The other 4 DNA nucleotides are converted from their RNA counterparts by ribonucleotide reductase. This implies there was a stage in evolution where DNA used uracil instead of thymine [41].

Thymidylate synthase follows 2 distinct patterns of evolution in the fully sequenced Chloroflexi genomes. All the *Dehalococcoides *have both ThyA and ThyX. This must be a derived state since one of the enzymes must have arisen before the other. It is possible that LUCA contained both of these enzymes, but very few species retain both of them. In many cases horizontal transfer displaces one with the other instead of retaining both as can be seen by looking at the distribution of these enzymes on a species tree (data not shown). It is far more likely that at least one of these enzymes is the result of a later horizontal transfer to the *Dehalococcoides*. The rest of the Chloroflexi contain only ThyX. However, this ThyX contains a domain duplication. This duplicated version of the protein has not been characterized, but is present in a few other species. It is possible that LUCA had a duplicated ThyX and the rest of the species have lost a domain, but this is clearly less parsimonious than this form of ThyX being derived.

We postulate that LUCA was Chloroflexi-like with a u-DNA genome. One of the first major branching points in the modern tree of life would be the origin of thymidylate synthase. All u-DNA genomes would eventually be out competed by t-DNA genomes in similar niches. Any u-DNA genome that received thymidylate synthase from a horizontal transfer should be all right since they already had all the machinery necessary for dealing with u-DNA. They would out compete similar u-DNA species. The distribution of thymidylate synthase in the Chloroflexi can only be explained by multiple horizontal transfers. It is possible one of the thymidylate synthases in *Dehalococcoides *represents the ancestral form and there were two later displacements (or a displacement and duplication event). Distinguishing between these scenarios is very difficult as an ancient horizontal transfer so close to the root of the tree would mimic vertical descent. Unfortunately all the sequence trees constructed have low bootstrap values for the critical edges, so we cannot conclude that each of the 3 different thymidylate synthases in the Chloroflexi are the result of horizontal transfer (data not shown). Therefore this might not be a falsifiable hypothesis, but it certainly is an interesting idea.

One could argue we have excluded the root from the ancestor of the Archaea and Firmicutes based on the presence of a derived PyrD, but have come up with an odd scenario to justify a rooting we like that has a derived thymidylate synthase. The major difference is that PyrD 1B appears to be present in almost every Archaeal and Firmicute genome, including the apparently deep branching Thaumarchaeota and Korarchaea, which makes horizontal transfers after their last ancestor unlikely. It is very unlikely that the history of thymidylate synthase in the Chloroflexi involved no horizontals transfers. There is also ample evidence that ThyX and ThyA frequently replace each other through horizontal transfers. It is reasonable for the Chloroflexi to have some derived traits even if they represent the most ancestral branch of the tree of life, as long as these traits appear to be the result of later transfers as in this case. Even with the correct rooting, it might not be possible to reconstruct LUCA because of later horizontal transfers, but this is a good example of how one can tell when a derived trait has been transferred to an ancient group.

There is growing evidence that the Archaea are derived from the Gram-positive Bacteria [7,12,13,24,31]. However there is still disagreement on whether that Gram-positive ancestor was a member of the Firmicutes or Actinobacteria (which is why the Archaea are placed ambiguously in Figure 6), as well as the source of selective pressure that was great enough to give rise to a novel superkingdom. Serious objections have been raised to the possibility that Archaea are derived from Bacteria based on differences in DNA replication machinery [42], but our own analysis suggests this divide is not as vast as some have suggested (in preparation). But that is beyond the scope of this work.

One of the corollaries of a rooting near the Chloroflexi is that the first true cells had two membranes. This may seem counterintuitive, but is actually well explained by Cavalier-Smith's obcell theory [19]. The idea is that the first organisms were cells that had nucleozymes anchored by short hydrophobic peptides to the outside of the membrane. In other words life started on the outside of cells, not inside them. This gets around the difficulty of forming transmembrane pores, a major difficulty in the RNA world. Heredity would still be based on the division of membranes, just as it is now. If two obcells were to fuse, the result would be a double membrane protocell. Very little follow-up has been done to test the plausibility of the obcell theory. We hope the additional evidence for a Gram-negative root provided in this work motivates other to investigate this idea further. There are currently about a dozen sequenced Eobacterial genomes compared to the hundreds of proteobacterial genomes discussed above. More research into the genomics of these possibly early branching bacteria could bring many obscured details about LUCA into the light.

## Methods

The multiple structural alignment in Figure 1A was performed using the MUSTANG webserver[43]http://www.cs.mu.oz.au/~arun/mustang/ and the one in Figure 3 was performed using the CE-MC webserver [29]http://pathway.rit.albany.edu/~cemc/. We used different alignment algorithms for these 2 data sets because in each case one program gave a higher quality alignment than the other. Pairwise structural alignments were performed using FATCAT [37]. Molecular graphics images were produced using the UCSF Chimera package [44].

The phylogenetic tree in Figure 5 was constructed using Phyml [45], packaged as part of Geneious http://www.geneious.com/. The tree was built from the multiple alignment of PRK07259 in the NCBI Protein Clusters Database [34]. The tree was drawn and colored using FigTree http://tree.bio.ed.ac.uk/software/figtree/.

The data used to generate Table 1 was taken by looking at the most populated clusters for each elongation factor in the NCBI Protein Clusters Database [34]. EF-Tu statistics were calculated using the sequences in PRK00049, PRK12735, and PRK12736. EF-1 sequences are from PRK12317. EF-G sequences are from PRK13351, PRK12740, PRK12739, and PRK00007. EF-2 sequences are from PRK07560. Only sequences that had both the RG(IV)T and PGH motifs were used to calculate insertion lengths, as these are then only cases that can be unambiguously compared without actually aligning the paralogs.

## Competing interests

This work is a critical evaluation of the work done by Lake *et al*., so there is an academic competing interest.

## Authors' contributions

REV conceived the study and analyzed the data. PEB assisted in writing the manuscript.

## Reviewers' comments

### Reviewer's report 1

*Greg Fournier, Department of Molecular and Cell Biology, University of Connecticut*, *Storrs, CT 06269-31258, USA (nominated by J. Peter Gogarten, Department of Molecular and Cell Biology, University of Connecticut, Storrs, CT 06269-31258, USA)*

In this article, Valas and Bourne attempt to resolve two disparate indel-based rootings of the tree of life proposed by Cavailer-Smith [7] and Lake [14], respectively. In each case, the presence of indels is used as a polarizing character for pairs of paralogous genes that duplicated before the LUCA, allowing for the exclusion of the root from particular branches of the tree. Using different sets of genes containing indels, Cavalier-Smith argues for a root within the Actinobacteria, while Lake argues for a root within the Firmicutes. The authors determine that the work presented by Lake *et al*. [14] does not necessarily exclude the rooting reported by Cavalier-Smith, thus supporting a root within the Actinobacteria, near the Chloroflexi.

#### Author's response

This is incorrect. Cavalier-Smith is not arguing that the root is anywhere near the Actinobacteria. He is arguing for a Gram-negative root and the Actinobacteria are Gram-positive. Lake *et al*. are also not arguing for a root within the Firmicutes either. We realize this is a confusing subject since each group is referring to non-standard higher level taxa. We have tried to be more explicit about the traditional vs non-traditional names as well be clear as to the number of membranes each of these groups has. It is vital to understand that Cavalier-Smith's root is based on multiple types of polarized evidence including but certainly not limited to indels.

Various methods exist for rooting the tree of life, primarily either using polarizing characters, or reciprocal rooting of paralogs. While the authors mention paralog rooting as an alternative method, their claim that it is not "self consistent" is an overstatement, as the supermajority of paralogs used in these analyses support a rooting within the bacterial stem, with a few others showing weaker support for a root at an undertermined position within the bacterial domain. None support a rooting within either the archaea or eukarya. That being said, the authors' stated objective is to root the tree relying only on indel-based evidence, and it is only fair to evaluate their conclusions solely within that context.

#### Author's response

We actually disagree with the statement that this work should be judged on indels alone. Our goal is to root the tree using any data possible. This work is just an evaluation of Lake *et al.'s *works on indels, but its important to remember the context of the argument rests on other data sources. We do not think we have overstated the inconsistencies created from paralog rooting. In the most comprehensive search for informative paralog rootings the true supermajority (137 out of 154) were inconclusive because they made both the Archaea and Bacteria polyphyletic due to horizontal transfer [5]. Of the 17 remaining paralogs sets 9 supported the rooting between the stem Archaea and Bacteria, 7 supported rootings within Bacteria, and 1 supported a rooting within the Archaea. The authors advised to use caution when accepting the rooting between the superkingdoms, because it is consistent with the tree long branch attraction would cause. In reviewing their own work the authors say "Large-scale search of anciently duplicated genes did not bring any consensus"[46]. The reason paralogs rooting is not self consistent is because there are many reasons why a sequence based tree will not reflect the evolution of cells. We hope the moral of this work is that structure is an untapped resource in rooting the tree.

The authors correctly mention that the quality of alignment is the major limitation to indel-based rooting approaches, and that the addition of structural information greatly improves the reliability of the method. However, even with the additional confidence that observed indels are real (and not alignment artifacts), their utility as polarizing characters still varies greatly based on the length and context of the indel. For example, the support for excluding the root from the archaeal/eukaryal grouping [11] shown in Figure 1 consists of a large region of protein sequence (14 AA) within EF-2 corresponding to a discrete helical surface structure within the protein. Compared to the homologous regions of EF-G and EF-Tu, it is clear that this is a derived, polarizing character. Indel evidence used for excluding the root from within bacterial groups seems to be far weaker in both hypotheses being compared, as small indels are more likely to be the result of convergence.

#### Author's response

We completely agree that larger indels make better phylogenetic markers and that our evidence excluding the root from Archaea and Eukaryotes is stronger than its placement within Bacteria. However, some still claim the root is within either of these groups [1,47] or between them (see Eugene Koonin's review below). In our opinion reaching a consensus on a root within the Bacteria would be a big step forward.

While the authors clearly show that the evidence provided by Lake et al. is insufficient to exclude the root from near the Chloroflexi based on their improved analyses, there is a logical fallacy at work in their conclusions. "We show that all four of these arguments have flaws and that is evidence that Cavalier-Smith's rooting is probably correct" is clearly a false dichotomy, as lack of evidence for the former does not correspond to any increase of evidence for the latter. The authors should have seriously considered (or at least discussed) the third possibility that there is simply not enough indel-based information for a reliable rooting of the tree of life (except for its exclusion from the archaeal/eukaryal branch).

#### Author's response

We disagree. The very nature of exclusive rooting is to prove a branch of the tree has a derived trait. Therefore, it is impossible to ever truly prove a rooting using this method. If one cannot exclude a root using more and more data our confidence in that rooting should increase. Every argument that could (or has been claimed) to exclude the root form the Chloroflexi but does not can be taken as evidence that rooting is correct. To us this is the first real independent test of Cavalier-Smith's hypothesis. We never claim that indel-based data are enough to root the tree. In fact we claim the opposite, since our PyrD quaternary structure argument goes against what some of the indel data implies. Polarized indel arguments will be limited in nature since they require universal paralogs, but their might be many polarizible transition in quaternary structure. The position that there is not enough polarizing data to root the tree was certainly defendable before this work because there were numerous disagreements in the data. We have resolved all of these, so for now it seems there is enough polarizing data to reliably root the tree. The 4 polarized arguments presented here are not enough to root the tree reliably on their own. Our point is that independent lines of reasoning are beginning to converge on a single rooting. Its time to test (attack) Cavalier-Smith's rooting using every piece of reliable data out there until it breaks. Then we think it would be worth discussing the possibility that there is not enough polarizable data to root the tree of life.

An interesting and novel rooting approach is also presented, based on the quarternary structure of the PyrD enzyme. Mapping the phylogenetic distribution of each type of enzyme (monomer, homodimer, heterotetramer) using the NCBI Protein Clusters Database, the authors show that the most parsimonious path for the evolution of these types (i.e., one of increasing subunit complexity) effectively excludes the root from being within the archaea, the firmicutes, or their most recent common ancestor, and thus requires it to be within a bacterial non-firmicute group (which contain the monomeric type, PyrD2). However, a preliminary phylogenetic investigation shows that PyrD homologs which are present as a homodimer (PyrD1A) are clearly a derived group within the heteratetramer (Pyr1B) set most closely related to the bacillus group within the firmicutes. Therefore, the presented parsimonious model of subunit evolution cannot be made to agree with any possible rooting of the tree. A more extensive phylogenetic analysis involving all PyrD homologs is clearly needed before this character can be used to exclude the root from any part of the tree.

#### Author's response

It is hard for us rebut a tree that we have not seen but here goes. Our hypothesis on quaternary structure evolution is not contradicted by our own analysis of the PyrD tree, using HisA as outgroup (data not shown). We do not see evidence that PyrD 1A is clearly derived from PyrD 1B. Even if we did, we would still argue that result could be an artifact. All 3 PyrD families are going to be under very different selective pressures since they each have different protein-protein interaction sites. This is a case where it would be completely possible for PyrD 1B to evolve rapidly out of PyrD 1A but look exactly as you described in the sequence tree. It is possible that PyrD 1A is derived from PyrD 1B, but until one provides an outgroup that explains the origin of the heterotetramer we are not going to find a sequence argument very convincing here.

### Reviewer's report 2

Purificación López-García, Unité d'Ecologie, Systématique et Evolution, UMR CNRS 8,079, Université Paris-Sud, bâtiment 360, 91405 Orsay Cedex, France

This work is a reanalysis of several conserved paralogous genes used by Lake et al. to place the root of the tree of life based on indel sharing that were in apparent disagreement with a rooting proposed by Cavalier-Smith between the Chloroflexi (Eobacteria) and the rest of bacterial + archaeal groups. A key factor to attempt such analyses is the quality of the alignment. Structural alignment data shows that the analysis those paralogous genes based on indels yields results that would be compatible with the rooting proposed by Cavalier-Smith.

This work is interesting as it shows some of the drawbacks that can be linked to this kind of indel analysis, particularly in what concerns ambiguous alignment and convergence. Yet, rooting the tree of life is a difficult task and there is possibly too little information left in ancient duplicated genes that allows answering that question with meaningful statistical support. From the four sets of genes studied here on a structural basis, two of them are discarded as unable to provide polarizing indel information: S12/RpoC because they may not be homologous, and Hsp70/MreB, which are inconclusive. The alignment of EF-G/EF-Tu proteins is ambiguous at the indel region used for polarization of character states, although quaternary structure-based alignment was not possible. Only two couples of paralogs yield what might be useful information according to Valas and Bourne, the HisA/HisF and PyrD/HemE.

#### Author's response

We do not consider the PyrD/HemE indel argument to be robust, but we reach the same conclusion using our polarization of quaternary structure. We also think the insert in GyrA robustly excludes the root from within the Actinobacteria, but there was no need to reanalyze that result here as with the other indels. We also accept the conclusion the EF-G/EF-Tu indel despite the problems with aligning these sequences.

Though of interest, one can wonder whether this information is enough to confidently exclude the root of the tree of life from everything outside the Chloroflexi. In addition of the alignment quality, there are other factors that can be of crucial importance here. One is convergence, as rightly pointed out by the authors, and the other is horizontal gene transfer (HGT). HGT is but very briefly mentioned here to exclude the possibility that PyrD has been transferred between archaea and bacteria recently. However, other protein genes, notably gyrase and Hsp70 genes are very likely cases of HGT from bacteria to archaea. Despite so, they are included in this kind of indel work. Careful phylogenetic analyses should be done for all the genes that are included in these attempts so that only vertically inherited genes are used. The possibility of important HGT levels between Firmicutes and/or Actinobacteria and archaea is not discarded. Finally, other problems such as hidden paralogies or even selective paralog loss cannot be excluded.

#### Author's response

It is true that everyone of these factors can mislead an indel analysis. We are mainly trying to improve the alignment step for indel sets that appear to meet these criteria to a reasonable level. Horizontal transfer of Hsp70 is irrelevant since this indel is not polarizable using an outgroup regardless of its distribution within species. Gyrase has probably been horizontally transferred, but it is clear from looking at sequence alignments that the Actinobacterial insert has not been transferred in a way that could confound the results. Horizontal transfers do not necessarily destroy an indel argument, we just need to be careful about whether they actually affect the conclusion or not. If a derived trait is horizontally transferred to ancient group the early branching members of that group will be unaffected. Unless there is a selective sweep or poor sampling we should be able to identify these cases. One also needs to keep in mind that horizontal transfers are defined by our assumption of what the correct tree is. This paper challenges the traditional rooting between the Archaea and Bacteria so many horizontal transfers presented in the literature may actually be better explained by vertical inheritance in this model. Loss is trickier since inferring where a loss as opposed to a gain occurs requires independent lines of polarization. We feel we are being very conservative in only really accepting 3 indel polarizations that seem robust to these sources of noise.

Another comment is that the fact that these results are compatible with a rooting between Chloroflexi/Eobacteria and anything else does not necessarily imply that the root is actually close to these organisms. First, more than 50% of the bacterial phylum-level groups correspond to candidate divisions without cultivated members. A similar trend occurs in archaea. Therefore, there is a considerable ignorance about the indel distribution in at least half of the bacterial diversity. Also, though the most parsimonious scenarios appear more likely to us, this is not proof that evolution proceeds that way. A cautionary vision should be held in this regard.

#### Author's response

We agree that this work is certainly not the last word on the rooting of the tree of life. As discussed above we feel this work is a good test of independent lines of reasoning to Cavalier-Smith's Eobacterial rooting. For now his hypothesis seems to be the one to beat. We are open to new data may change the picture, but the point of this paper is that the present data does not contradict itself in the manner it appears to in the current literature. There is an important difference between parsimony and polarization. To us parsimony can be used to analyze events where gain and loss have nearly equal probabilities, while polarizations imply that one direction would evolve more easily than the other. Consider the example of the proteasome discussed in detail in [7]. A parsimony argument would be that the 20s proteasome is the result of a duplication so a non duplicated structure must precede it. The polarization argument involves considering the structure and function of proteasomes as well as the fitness of the intermediates to argue that evolution towards the 20s proteasome is much more plausible than the reverse direction. There are probably many cases where evolution has not been parsimonious, and we do not think parsimony is a safe or productive assumption. However, there appears to be many polarizable transitions and hopefully there are many more waiting to be discovered. If the new data continues to support the Eobacterial rooting then our confidence in it will increase.

### Reviewer's report 3

Eugene V. Koonin, National Center for Biotechnology Information, National Library of Medicine National Institutes of Health, Bethesda, Maryland 20894, USA

This manuscript addresses very important issues of the position of the primary divide among eukaryotes and, by implication, the relationships between archaea and bacteria, and the nature of the LUCA. The discussion is presented in somewhat obsolete terms of the "root of the tree of life". As there is no such thing as a single "tree of life", speaking of a single root is somewhat misleading but the central question is nonetheless meaningful and crucial.

#### Author's response

We do not believe that the issue of the nature of the tree of life has been settled. It is certainly true that recent work has shown that there is too little signal to resolve the tree of life using sequence alone [17,18], and that many genes have histories distinct from the species in which they reside. However, it is limiting to assume that the only data useful for building the tree of life is sequence data, and that additional data will be unworthy in this pursuit. We are aware that tree representations have many shortcomings, but we still believe it is the single best metaphor to describe the major events in evolution. We are working with a novel data source to argue the tree of life is realer than studies in genomics have led us to believe (in preparation). The phrase "root of the tree of life" may not be as accurate as "the first polarizable transition between extant groups", but it certainly rolls off the tongue better.

Without going into the minute details of the analysis of indels in specific protein families, I will state my firm view of this issue. The nature of the primary divide in prokaryotes – and actually among all cellular life forms is clear, and it is between archaea and bacteria. This view is supported by the fundamental differences between archaeal and bacterial systems of DNA replication, core transcription, translation, and membrane biogenesis – essentially, all central cellular systems (not just the replication system as noted in the present paper). I believe these differences are sufficient to close the "root debate" (regardless of the appropriateness or lack thereof of the very notion of a root in this context) and to base analyses and discussions aimed at the elucidation of the nature of LUCA on that foundation.

#### Author's response

One cannot disagree with the fact there are vast differences between the Archaea and Bacteria, and we are well aware of the details of that argument. We believe that none of these differences are as great they appear at first glance, and we are working on a scenario to detail the transition between the Bacterial and Archaeal DNA replication system. It certainly makes sense the greatest splits in the tree would be the most ancient. However, we are proposing that the alternative hypothesis that a unique event in evolution occurred between the Bacteria and Archaea must be taken seriously. A rooting between the Archaea and Bacteria would imply the first Bacteria were Gram-positive. As Cavalier-Smith pointed has pointed out no one has adequately described how the transition from a Gram-positive to a Gram-negative bacterium could occur [7]. So the rooting you assume to be true has its own problems too. Cavalier-Smith has already proposed a detailed scenario that covers many of these transitions you mention [24] and Lake *et al*. have recently discussed the issue of membrane biogenesis [12]. That said there are aspects of both of these hypotheses on the origin of the Archaea that we feel are incomplete. At this point it seems reasonable to keep an open mind about the root, but this work argues against all evidence that has ever been used to support a Gram-positive Bacterial rooting. Until there is a scenario that describes all the major transitions starting from the root that has been robustly tested by multiple lines of evidence we suggest this debate is not over. We think the data presented here offers a compelling reason to continue to look at the details of the Gram-negative rooting.

Indel analysis is a legitimate method of phylogenetic inference but is seriously hampered not only by horizontal gene transfer but, more importantly, by the possibility of homoplasy, that is, independent insertions in the same region of homologous proteins. The contradicting macro-phylogenetic inferences made by Lake, Cavalier-Smith, Gupta and others using this approach serve to illustrate the point. The use of structures to corroborate indels is, of course, a good idea in principle but changes very little substantially. Somewhat parenthetically, I find it strange that the alignments of this paper, aimed primarily at clarification of the relationship between archaea and bacteria, include only bacterial and in some cases eukaryotic proteins.

#### Author's response

We disagree that this changes nothing substantial. Every single disagreement between these different groups is, in our view, caused by bad alignments. It appears to us the only substantial difference between Gupta and Cavalier-Smith's phylogenies were based off of Gupta's polarization of the Mreb/Hsp70 indel, which we have demonstrated is inconclusive. This work has significantly improved the quality of the alignments and resolved all contradictions within these data sets. We hope this begins to forge a consensus between them, and stimulates brain storming on how the systems you mention above could undergo such dramatic changes. In our opinion, one must deal with the differences between the macro-phylogenies one detail at a time instead of assuming they are a quixotic pursuit. If these macro-phylogenies are truly incompatible it should not be possible to get them to converge on a single tree as we believe we have done here. Again, it is true that transfers and convergent evolution complicate indel analysis, but they do not invalidate it *a priori*. Multiple transitions are necessary to make a tree robust to these problems. We have been very stringent in accepting an indel as informative, so we are confident our conclusions are not a result of these factors. Our analysis of PyrD demonstrates that our conclusion is not the result of horizontal transfer, and the clustering of PyrD 1B shows it is not the result of convergent evolution. The distribution of this derived structure across a Gram-positive group and the Archaea must be considered seriously as evidence that the root might not be between the Archaea and Bacteria. We agree that it would be ideal to use more Archaeal structures in our alignments. However, we are limited by currently available crystal structures. At the present time it appears very few people besides us are really considering structure to be a useful tool in studying the major events in evolution. Therefore there is no directed effort aimed at widely sampling the same structure across the tree of life. We believe this paper shows that structure has a role to play in every aspect of studying the events that separate the major taxa. The landscape of the continent of genomics is being filled in rapidly, but the continent of protein structure, especially quaternary structure, lags far behind. We are optimistic that structure may still contain enough signal to resolve a single backbone to the tree of life where sequence has failed.
